# Exploring unnecessary invasive procedures in the United States: a retrospective mixed-methods analysis of cases from 2008-2016

**DOI:** 10.1186/s13037-017-0144-y

**Published:** 2017-12-18

**Authors:** James M. DuBois, John T. Chibnall, Emily E. Anderson, Heidi A. Walsh, Michelle Eggers, Kari Baldwin, Kelly K. Dineen

**Affiliations:** 1 Division of General Medical Sciences, Washington University School of Medicine, 660 S. Euclid Avenue, Campus Box 8005, St Louis, MO 63110 USA; 2 Department of Neurology & Psychiatry, Saint Louis University School of Medicine, 1438 S. Grand Blvd, St. Louis, MO 63104 USA; 3 Neiswanger Institute for Bioethics & Health Policy, Loyola University Chicago Stritch School of Medicine, 2160 S. First Avenue, Maywood, IL 60153 USA; 4 Creighton University, School of Law, 2500 California Plaza, Omaha, NE 68178 USA

**Keywords:** Unnecessary procedures, Medical misconduct, Mixed methods, Standard of practice

## Abstract

**Background:**

Unnecessary invasive procedures risk harming patients physically, emotionally, and financially. Very little is known about the factors that provide the motive, means, and opportunity (MMO) for unnecessary procedures.

**Methods:**

This project used a mixed-methods design that involved five key steps: (1) systematically searching the literature to identify cases of unnecessary procedures reported from 2008 to 2016; (2) identifying all medical board, court, and news records on relevant cases; (3) coding all relevant records using a structured codebook of case characteristics; (4) analyzing each case using a MMO framework to develop a causal theory of the case; and (5) identifying typologies of cases through a two-step cluster analysis using variables hypothesized to be causally related to unnecessary procedures.

**Results:**

Seventy-nine cases met inclusion criteria. The mean number of documents or sources examined for each case was 36.4. Unnecessary procedures were performed for at least five years in most cases (53.2%); 56.3% of the cases involved 30 or more patients, and 37.5% involved 100 or more patients. In nearly all cases the physician was male (96.2%) and working in private practice (92.4%); 57.0% of the physicians had an accomplice, 48.1% were 50 years of age or older, and 40.5% trained outside the U.S. The most common motives were financial gain (92.4%) and suspected antisocial personality (48.1%), followed by poor problem-solving or clinical skills (11.4%) and ambition (3.8%). The most common environmental factors that provided opportunity for unnecessary procedures included a lack of oversight (40.5%) or oversight failures (39.2%), a corrupt moral climate (26.6%), vulnerable patients (20.3%), and financial conflicts of interest (13.9%).

**Conclusions:**

Unnecessary procedures usually appear motivated by financial gain and occur in settings that have oversight problems. Preventive efforts should focus on early detection by peers and institutions, and decisive action by medical boards and federal prosecutors.

## Background

When unnecessary invasive procedures are performed, patients lose time and money and may be caused mental distress, physical harm, or death [1, 2]. The following cases from the United States illustrate the direct harms caused to patients by unnecessary procedures. A Louisiana physician performed unnecessary cardiac catheterizations and stents, angiograms, and angioplasties on approximately 310 patients over at least 5 years; one patient died and hundreds of others suffered harm [3, 4]. A Florida ophthalmologist misdiagnosed patients with wet macular degeneration to justify unnecessary laser eye surgery. He falsified patient records and ordered unnecessary diagnostic tests to support his fabricated diagnoses [5]. A South Dakota surgeon repeatedly performed medically unnecessary spinal surgeries for profit, resulting in patient injury and death. Despite complaints filed by his medical staff and former patients and their family members, he retained his privileges and continued to practice for many years [6, 7].

Physicians who perform unnecessary procedures may face medical board discipline, loss of clinical privileges, lawsuits from harmed patients or third-party payers, exclusion from state or federal programs, state or federal criminal charges, and other possible repercussions with penalties ranging from monetary damages to medical license revocation to incarceration [1, 8, 9]. A single act can result in myriad judicial and quasi-judicial actions. The physicians in the cases cited above were defendants in criminal prosecutions, False Claims Act and class action litigations, and administrative proceedings before state medical boards. Penalties included prison, the imposition of monetary damages and fines, and loss of licensure [3, 7, 10–13].

The study reported in this paper is part of a larger project to understand causal factors associated with significant professional breaches in medicine such as running an opioid “pill mill,” [14] inappropriately touching patients during exams, [15] and performing unnecessary surgeries for profit. We have focused on high-profile cases of professional breaches that directly cause significant harm to patients. Fortunately, such breaches are relatively rare [8].

Nevertheless, deviations such as unnecessary invasive procedures are serious and often high-profile. They threaten the reputation of the medical profession. Training programs and oversight bodies should strive to prevent, identify, and mitigate the occurrence of unnecessary invasive procedures. In order to achieve these goals, it is important to know what factors enable unnecessary procedures to occur.

Very little is known about unnecessary invasive procedures. Lawyers have written about the subject, noting the problems caused for patients and federal funding of healthcare [2, 16–18]. They have observed that complexity of determining medical necessity [19, 20], “gray areas” in the standard of care, [17, 20] and fee-for-service payment structures play a role in unnecessary procedures [21, 22]. Studies of disciplinary actions against physicians rarely explore unnecessary procedures in detail; rather, such cases appear buried within the broad categories of fraud or medical malpractice. Further, most such studies limit their scope to documenting frequencies and associations with a small number of variables that may or may not be causally related to cases, such as board specialty and physician age [23]. Other studies that have explored potential predictors of serious professional breaches in medicine, such as prior investigations by medical boards [24] or unsolicited patient complaints, [25–27] provide some insight into the general problem of deviance in medicine, but do not provide detailed descriptions or causal analyses of unnecessary procedures.

No published study has adopted a motive, means, and opportunity (MMO) framework to explore why and how cases of unnecessary invasive procedures occur [28, 29]. While motivation may seem straightforward in such cases (financial profit in a fee-for-service system), questions remain: Were individuals subject to disciplinary action due to honest disagreement with standards of care? Did patient demands play a role? Did physician impairment play a role (e.g., substance abuse or mental disorders such as mania)? Are unnecessary procedures more commonly performed on older or particularly vulnerable patients? Regarding opportunities, are unnecessary procedures more strongly associated with certain practice environments such as academic medicine or solo practices? Do unnecessary procedures require collaboration from co-workers? From a purely descriptive perspective, what happens to physicians who perform unnecessary procedures? What percentage continue practicing medicine, and when they do, following what kinds of interventions?

Little is known about unnecessary procedures in part because cases involving deviations from the standard of care in medicine are difficult to study. First, researchers could no sooner conduct a randomized controlled trial of interventions aimed at inducing unnecessary procedures than they could conduct a randomized controlled trial of the effectiveness of parachutes [30]. Rather, like other serious professional breaches, unnecessary procedures need to be studied using an exploratory, “causes of effects” model: Starting with the effects (an unnecessary procedure), one must examine cases using a theoretical framework to develop an exploratory causal theory [31]. This requires that cases are readily identifiable and that a rich array of case characteristics are reported (e.g., characteristics of the physician and the practice environment). Thus, a second obstacle to studying serious professional breaches in medicine arises from the fact that those who deviate from standards of practice are often effective concealers, leaving most cases unreported [15]. Third, when cases are reported to oversight bodies that maintain public records such as the National Practitioners Database, they are often reported using vague terminology (such as “not applicable” or “other”) [24]. Finally, accurately reported cases are typically de-identified and lack detailed case characteristics because National Practitioners Database policy prohibits the public (including researchers) from accessing identifiable records [32].

Exploratory, causes-of-effects designs are limited in their ability to control for possible confounding variables and accordingly cannot demonstrate causality. Nevertheless, they can generate rich descriptions and exploratory causal models of complex social phenomena such as unnecessary procedures. Previous work by our team found that such work is feasible for three reasons. First, large convenience samples of cases can be obtained by culling publicly available records from medical boards and court cases; second, a wide variety of variables can be reliably coded to generate rich descriptions of cases and causal theories; and third, the MMO framework provides a useful theoretical lens for analyzing the complex causal factors that contribute to the occurrence of serious professional breaches [14, 15, 33]. The present study applied this approach to the study of invasive procedures such as stents or surgeries that were deemed unnecessary by medical boards, courts, or federal prosecutors.

## Methods

We used a mixed-methods design that involved five key steps: (1) systematically searching the literature to identify cases of unnecessary procedures; (2) identifying all medical board, court, and news records on relevant cases; (3) coding all relevant records using a structured codebook of case characteristics; (4) analyzing each case using a MMO framework to develop a causal theory of the case; and (5) identifying typologies of cases through a two-step cluster analysis using variables hypothesized to be causally related to the performance of unnecessary procedures. We describe each step below.

### Inclusion criteria and identification of cases

We focused on cases that met the following six inclusion criteria: (a) the procedure was performed by a physician; (b) case occurred in the U.S.; (c) case was reported between January 1, 2008 – May 27, 2016; (d) case involved the performance of invasive procedures (thus we excluded, e.g., prescription of oral medications and imaging tests); (e) the procedures were deemed by colleagues or investigators to be medically unnecessary; and (f) the case was described in at least 5 independent records. The last criterion was established to ensure the availability of adequate information about the events, the physicians, and their practice environments.

To identify cases, we used the LexisNexis Law database, which archives statutes, case judgments, and legal opinions, and provides access to medical board and other regulatory documents, as well as U.S. newspaper articles. With the assistance of two law librarians, we developed a Boolean search strategy, which was used to search LexisNexis Law.^1^ The search returned 7832 records from the U.S.; 236 described cases that appeared to meet the six inclusion criteria described above, thus meriting further investigation. The project manager thoroughly reviewed the 236 records and found the names of 301 physicians who were involved (25 records listed multiple protagonists’ names). Of these 301 physicians, 222 were excluded as ineligible: 81 described cases of billing-only fraud or kickback schemes; 53 involved cases of unnecessary tests or non-invasive treatments; 18 involved cases of negligence; 18 detailed the involvement of co-conspirators in cases that were already deemed eligible; 18 cases lacked adequate literature to enable content analysis; 13 articles involved cases that were too old or too recent (i.e., the case had not yet been resolved either through board, criminal, or civil action); 12 cases were too ambiguous or the protagonist was exonerated; 6 cases involved non-physician protagonists; and 3 cases did not occur in the U.S. We investigated the remaining 79 eligible cases. In cases that involved multiple physicians, we focused on the physician who actually performed the majority of unnecessary procedures; thus, in our results, demographic variables describe just one physician per case.

### Identifying all Records of Cases

Once we identified a physician who performed unnecessary procedures, all records pertaining to the case were identified by conducting a thorough search using the physician’s name in a variety of databases including: LexisNexis Law, Google, state medical board websites, state circuit court access sites, HealthGrades, the American Board of Medical Specialties’ Certification Matters website, and the U.S. Office of the Inspector General’s exclusions website.

### Coding case characteristics

We engaged in descriptive coding of case characteristics. We developed an Excel codebook that defined variables and provided a dropdown menu for scoring each variable. The codebook contained 57 categorical case variables: 13 variables characterized the event (the number of unnecessary procedures, the kinds of procedures performed, and the patients involved); 22 variables characterized the physician and the work environment; and 22 variables characterized the response to the unnecessary procedures (the whistleblowers, investigations, charges, and penalties). The codebook also contained continuous variables on the year the case ended, the number of diverse records consulted, and additional deviations that accompanied unnecessary procedures.

We coded in two rounds. Most variables were deductive in that they were based on our systematic literature reviews and past research projects on cases of professional breaches [14, 15, 34, 35]. However, some variables—such as specific fraud charges, relationships to industry, and the dollar amounts billed—were inductive, arising from the process of coding the cases. Because many variables were inductive, and some deductive variable definitions needed to be adapted to the unnecessary procedures cases (e.g., oversight failures look different in unnecessary procedures than in sexual abuse cases), it was necessary to code all cases twice: Once to refine the codebook and once to produce a final database.

Given the large quantity of literature on each case and the large number of variables, coding required approximately 20 h per case; accordingly, cases were coded by one of three case researchers. We controlled the quality of coding in multiple ways. Throughout the coding period, the research team met weekly, which provided the opportunity to discuss questions about code definitions and new codes. A PhD-level co-investigator (EA) read at least 2 articles on each case, examined the preliminary coding of all cases, and provided feedback to the case researcher. Disagreements regarding codes were discussed with the principal investigator (JD) before finalizing coding. After the initial coding was completed on the first 50 cases, the frequency with which case researchers used codes was compared statistically to identify any significant discrepancies, which led to further clarification of how some codes were interpreted and applied.

### Applying the MMO theory to each case

After case attributes were coded, case researchers were asked to apply the MMO theoretical framework to develop a theory of the case. This involved coding whether or not a series of individual motives and environmental factors appeared to causally contribute to the occurrence of the case. Within a legal framework, motive is defined as “an emotion or state of mind that prompts a person to act in a particular way…” ([36], p. 445). Accordingly, motives may include not only things one wants to obtain (such as money or fame), but also individual traits such as antisocial personality disorder, substance use disorders, and carelessness. We cannot know motives directly because they are psychological states; in this study, as in legal settings, it was necessary to infer motives from circumstantial evidence or statements by perpetrators [36].

Because most physicians have the means of performing unnecessary procedures (that is, they are licensed and authorized to perform and bill for procedures), we focused on environmental factors that provided opportunity. These included: Ambiguous practice guidelines or norms, oversight failure, lack of oversight, corrupt moral climate, vulnerable patients, and financial conflicts of interest (relationships with industry that actually appeared to enable the performance of unnecessary invasive procedures).

### Cluster analysis of cases to identify typologies

Two-step cluster analysis (IBM SPSS Statistics, version 24) was used to create clusters of cases. This procedure attempts to form clusters of cases that maximize homogeneity on the clustering variables within clusters and maximize heterogeneity on the clustering variables between clusters. With categorical variables, a log-likelihood measure applies a probability distribution to the clustering variables to determine distances between clusters. By comparing the values of the model-choice criterion (here, Schwarz’s Bayesian Information Criterion) across different solutions, two-step cluster analysis automatically determines the optimal number of clusters and evaluates the goodness-of-fit of the solution using a silhouette measure of cohesion and separation (poor = −1.0 to <0.2; fair = 0.2 to 0.5; good = > 0.5 to 1.0).

The cluster analysis utilized theory of the case variables, including traits and motives of the physicians and environmental factors. Traits and motives included suspected antisocial personality, poor problem-solving, financial gain, and ambition; environmental factors included ambiguous norms, oversight deficits (lack of oversight and/or oversight failure), corrupt moral climate at work, and financial conflict of interest. Several trait/motive and environmental variables were excluded due to low prevalence and/or failure to discriminate among cases (mental illness, carelessness, substance abuse, stress/job pressure, retaliation, vulnerable patients, and conflicting roles).

## Results

Seventy-nine cases met inclusion criteria. The mean (standard deviation, SD) number of documents or sources examined for each case was 36.4 (32.6), including 7.2 (8.8) legal documents and 29.6 (41.0) pages of medical board records. Cases occurred in 28 states, with the greatest number occurring in Florida (17.7%, *n* = 14), followed by California (8.9%, *n* = 7), New York, and Ohio (each 7.6%, *n* = 6); 24 other states accounted for ≤4 (5.1%) cases each.

Cases were described with respect to a taxonomy of 14 different kinds of professional deviations from standards of care in medicine that may have accompanied the unnecessary procedures. On average, cases involved 4.3 (1.8) different kinds of deviations. The most prevalent deviations (≥ 10%) accompanying the unnecessary procedures were financial fraud that was formally prosecuted under a fraud law (83.5%), patient informed consent violations (81.0%), procedural violations (27.8%), illegal activities outside of professional breaches (26.6%), physician failure to provide required oversight (22.8%), improper prescribing or violation of drug statutes (22.8%), conflict of interest violations (16.5%), practicing while impaired/incompetent (13.9%), and unjust treatment of patients (11.4%).

Table 1 presents descriptive results for the case event attributes. In a majority of cases, (53.2%), unnecessary procedures were performed across a period of 5 years or more and the dollar amounts billed were substantial (in the millions of dollars for nearly half the cases). A majority of cases (56.3%) involved 30 or more patients, and 37.5% involved 100 or more patients. Nearly three-quarters of physicians (73.4%) billed Medicare for unnecessary procedures.

**Table 1:** The number and kind of deviations, procedures performed, and patient characteristics

Deviations from Standard of Care		Procedure Performed	
Deviations >1 type	73.4%	Invasive cardiology	26.6%
Deviations in >1 environment	48.1%	Spinal fusion/surgery	15.2%
Repeated unnecessary procedures	97.5%	Other surgery	31.6%
Period of unnecessary procedures		Infusion	11.4%
< 1 year to <2 years	7.6%	Other	15.2%
2 to <5 years	39.2%	Patient Characteristics	
5 + years	53.2%	No. of patients: >30^a^	56.3%
Dollar amount billed		Patient age	
< 100,000	5.1%	Adult	22.8%
100,000–500,000	6.3%	Senior	21.5%
500,001–1 million	3.8%	Child	2.5%
1,000,001–2 million	7.6%	General, mixed population	53.2%
> 2 million	45.6%	Women explicitly targeted	2.5%
Unknown	31.6%	Racial minority explicitly targeted	3.8%
		Medicare billed	73.4%
		Cases involve patient deaths	31.6%

^a^Information on number of patients unavailable in 15 cases. The denominator used here is 64

Table 2 presents the frequencies of variables characterizing the physician and key elements of their work environments. In nearly all cases the physician was male (96.2%) and working in private practice (92.4%). Several variables split the population fairly evenly: 57.0% of the physicians had an accomplice, 48.1% were 50 years of age or older, 48.1% demonstrated antisocial personality traits, and 40.5% trained outside the U.S. The most prevalent single specialty was cardiology (26.6%); 27.8% of physicians, however, were in a specialty that involved surgery. Overall, physicians did not have significant industry relationships (e.g., 8.9% had speaking or consulting relationships). Relatively few evidenced severe mental illness (1.3%) or substance addiction (2.5%), but 11.4% evidenced significant personal problems during the time when they performed unnecessary procedures, such as undergoing a divorce or bankruptcy.

**Table 2:** Individual and environmental characteristics

Physician Description		Significant personal problems	11.4%
Age > 49 years old	48.1%	Poor professional skills	12.7%
Gender: Male	96.2%	Claimed cases as uniquely difficult	12.7%
Born outside the US	27.8%	Relationship to Industry^a^	
Trained outside the US	40.5%	Gifts	3.8%
Specialty		Consulting/Authorship/Speaking	8.9%
Internal/General	8.9%	Grants for education or research	3.8%
OB/GYN	3.8%	Ownership interest	8.9%
Psychiatry/Neurology	1.3%	Physician owned distributorship	2.5%
Pediatrics/Family	5.1%	Other relationship to industry	11.4%
Anesthesiology	6.3%	Workplace	
Other surgery/emergency/ENT	7.6%	Non-Academic, Private Practice	92.4%
Urology	3.8%	Physician practice size	
Cardiology/Interventional	26.6%	Solo	17.7%
Neurosurgery	10.1%	Small (2–3 physicians)	12.7%
Orthopedics/Surgery	10.1%	Large (≥ 4 physicians)	55.7%
Oncology	6.3%	Other/Unknown	13.9%
Other	10.1%	Physician ownership	
Board certified	70.9%	Solo	29.1%
Antisocial personality traits	48.1%	Joint	13.9%
Engaged in unrelated illegal actions	21.5%	Employee	45.6%
Evidence of severe mental illness	1.3%	Other/Unknown Motive	11.4%
Substance addiction	2.5%	Accomplice involved	57.0%

^a^Relationships to industry means that reports on the unnecessary procedures mentioned such relationships. The cases investigated occurred before the Physician Payments Sunshine Act was effective, so no publicly available database of relationships existed. Total percent with any kind of relationship was 24.1%

Table 3 presents descriptive findings for variables characterizing the response to the unnecessary procedures, including whistleblowing, the investigation, charges, and penalties. In about 35% of cases, opportunities existed to report suspicion that unnecessary procedures were being performed (e.g., other physicians observed the behavior), but action was not taken; in addition, in one-quarter of the cases, the unnecessary procedures were reported at some level, but the whistleblower was ignored. Physicians were charged under myriad fraud-related laws, including federal criminal charges (35.4%), false claims (34.2%), other federal fraud or abuse laws (15.2%), state criminal charges (15.2%), anti-kickback (13.9%), and civil monetary penalties law (12.7%). The majority of cases involved board, criminal, and civil investigations (57.0%–72.2%). Prevalent consequences to the physicians included financial penalties (70.9%), loss of job/professional opportunities (73.4%), discontinuing medical practice (58.2%), and loss of licensure (55.7%).

**Table 3:** Response to unnecessary procedures: whistleblowers, investigation, fraud charges, consequences

Whistleblowers		Specific Fraud Charges	
Missed opportunity to blow whistle	35.4%	False claims	34.2%
Whistleblower ignored	25.3%	Anti-Kickback	13.9%
Whistleblower type		Stark law	2.5%
Patient	16.5%	Exclusion statute	5.1%
Peer/Physician colleague	19.0%	Civil monetary penalties law	12.7%
Nurse or other staff	10.1%	Other federal fraud or abuse law	15.2%
Other	36.7%	Title 18 federal/criminal charges	35.4%
None/Unknown	17.7%	State criminal charges	15.2%
Investigation		Consequences^a^	
Board investigation	72.2%	Loss of licensure	55.7%
Criminal investigation	64.6%	Financial penalties	70.9%
Civil proceedings	57.0%	Prison/Probation or service	31.6%
Others were found guilty	34.2%	Mandated treatment or education	20.3%
		Discontinued practicing medicine	58.2%
		Loss of job/professional options	73.4%
		Increased oversight/monitoring	30.4%

^a^In 2 cases the physician died prior to sentencing

Table 4 defines the motives identified in cases and their frequency. The most common motives were financial gain (92.4%) and suspected antisocial personality (48.1%), followed by poor problem-solving or clinical skills (11.4%) and ambition (3.8%). All other variables appeared in fewer than 2% of cases or never. In 2.5% of cases, no motive was apparent.

**Table 4:** Frequencies and definitions of the apparent motives

Financial gain	Performing unnecessary procedures generated significant revenue well beyond standard medical practice. E.g., literature highlighted that the physician billed >$1 million for unnecessary spinal fusion surgeries.	92.4%
Personality disorder	Literature provided evidence of 2 or more DSM-V criteria for a diagnosis of antisocial personality disorder. E.g., physician arrested for unrelated matters, continued performing unnecessary procedures after a patient death, and showed a lack of remorse.	48.1%
Poor problem solving	Unnecessary procedures appeared due to poor knowledge of standards of practice or deficient clinical skills.	11.4%
Ambition	Unnecessary procedures appeared motivated by career ambition, e.g., to enhance stature in the field or within the institution.	3.8%
Mental illness	Literature mentioned diagnosis with a severe mental illness such as bipolar disorder, schizophrenia, or major depression, and this appeared to play a causal role in the unnecessary procedures.	1.3%
Carelessness	Evidence that unnecessary procedures occurred due to carelessness rather than intentional fraud or incompetence.	1.3%
Substance abuse	Substance use disorder appeared to causally contribute to the performance of unnecessary procedures, e.g., by impairing judgment or creating an increased need for cash to support addiction.	0%
Stress	Significant personal stress such as bankruptcy or divorce appeared to impair decision making.	0%
Retaliation	Unnecessary procedures appeared motivated by the perception that the system is unjust or in retaliation against an institution for causing personal harm.	0%
None	No motive could be identified or reasonably inferred.	2.5%

Table 5 presents the definition and frequency of environmental factors that provided opportunity for the unnecessary procedures. The most common environmental factors included a lack of oversight (40.5%) or oversight failures (39.2%), a corrupt moral climate (26.6%), vulnerable patients (20.3%), and financial conflicts of interest (13.9%).

**Table 5:** Frequency and definitions of variables that provided opportunity

Lack of oversight^a^	The environment did not afford the ordinary oversight of Centers for Medicare and Medicaid Services- or Joint Commission-mandated processes or Internal Auditing for billing. E.g., physician owned small outpatient surgery center.	40.5%
Oversight failure	Oversight mechanisms existed or should have existed, but were so deficient that opportunity for unnecessary procedures was established. E.g., unnecessary procedures were performed for 5 years amidst complaints.	39.2%
Corrupt moral climate	A corrupt moral climate contributed to the unnecessary procedures, e.g., institutional officials collaborated in or encouraged the procedures. This variable was not used in addition to oversight failure, but it could be a cause of a lack of oversight.	26.6%
Vulnerable patients	Patients belonged to a protected class (e.g., children or older adults) or had cognitive deficits, and this appeared to create opportunity for unnecessary procedures.	20.3%
Financial conflict of interest	The physician had relationship to industry (e.g., consulting contracts) and this appeared to contribute to unnecessary procedures.	13.9%
Ambiguous norms	The standard of practice was not well established, and this “gray area” created opportunity for unnecessary procedures.	5.1%
Other	An environmental factor, not listed above, appeared to create opportunity for unnecessary procedures.	3.8%
Conflicting roles	The physician played conflicting roles, e.g., treating physician and chair of the patient care review committee, and this created opportunity for unnecessary procedures.	0%
None	No environmental factor presenting opportunity could be identified or reasonably inferred.	13.9%

^a^80% (63/79) of cases involved either a lack of oversight or oversight failure, that is, some form of oversight problem

### Cluster analysis

A five-cluster solution was obtained, with a goodness-of-fit measure of 0.6, indicative of a good quality solution. Table 6 indicates the pattern of clustering variables among the five clusters. Cramer’s V, a measure of association among nominal variables (range = 0–1), was used to indicate strength of association between clustering variables and cluster groups. As the table shows, clusters were primarily defined by (1) financial gain, personality disorder, and oversight deficits; (2) poor problem solving and oversight deficits (with some financial gain); (3) financial gain, oversight deficits, and financial conflict of interest; (4) financial gain (with some personality disorder and financial conflict of interest); and (5) financial gain, oversight deficits, and corrupt moral climate (with some personality disorder).

**Table 6:** Cluster analysis results (*N* = 79)

Clustering Variables	Clusters					V^**a**^	n
	1 (*n* = 21)	2 (*n* = 9)	3 (*n* = 17)	4 (*n* = 11)	5 (*n* = 21)		
Traits/Motives							
Financial gain	100%(21)	66.7%(6)	100%(17)	81.8%(9)	95.2%(20)	.42	73
Personality disorder	100%(21)	22.2%(2)	11.8%(2)	45.5%(5)	38.1%(8)	.67	38
Poor problem solving	0%	100%(9)	0%	0%	0%	.99	9
Ambition	0%	0%	5.9%(1)	0%	9.5%(2)	.22	3
Environmental factors							
Oversight deficits	100%(21)	77.8%(7)	82.4%(14)	0%	100%(21)	.83	63
Corrupt moral climate	0%	0%	0%	0%	100%(21)	.99	21
Financial COI	0%	0%	47.1%(8)	0%	14.3%(3)	.53	11
Ambiguous norms	0%	0%	17.6%(3)	9.1%(1)	0%	.33	4
Summary of Clusters	1	2	3	4	5		
Financial gain	H	M	H	M	H		
Suspected antisocial	H	M	L	M	M		
Poor problem-solving	–	H	–	–	–		
Ambition	–	–	L	–	L		
Oversight deficits	H	M	M	–	H		
Corrupt moral climate	–	–	–	–	H		
Financial COI	–	–	M	–	L		
Ambiguous norms	–	–	M	L	–		
Seriousness rating	54.4 (7.9)	51.9(8.2)	45.6(11.7)	50.9(7.9)	47.9(10.8)		

^a^Cramer’s V was used to test the association of these nominal variables. Values above .35 are particularly important in discriminating among clusters, which is reinforced by *p*-values of < .05 for those Vs; values < .35 indicate weak relationships that did not discriminate significantly

Percentage is percent of cases within the cluster. The raw number of cases representing a variable appears in parentheses

*H* High (84–100%), *M* Medium (17–83%), *L* Low (1–16%); − = Absent

With respect to the type of procedures performed (see Table 1), Cluster 1 included all procedures, but with the highest prevalence (47.6%) associated with other (non-orthopedic) surgeries, followed by 19.0% infusion-related and 19.0% spinal fusion surgery (remaining procedures were <10%). Cluster 2 included all procedures except spinal fusion surgery: 22.2% infusion-related, 11.1% invasive cardiology, 33.3% other surgery, and 33.3% “other” procedures. Cluster 3 was heavily surgical (29.4% spinal fusion surgery, 29.4% other surgery), followed by invasive cardiology and “other” (each 17.6%). Cluster 4 was primarily invasive cardiology (54.5%), followed by spinal fusion surgery and other surgery (each 18.2%). Cluster 5 was also heavily invasive cardiology (47.6%), followed by other surgery (23.8%) and other procedures (14.3%).

With regard to the seriousness of performing unnecessary procedures, we created a summary seriousness variable composed of the number of professional and criminal consequences to the physician (see Table 1), the period of time during which unnecessary procedures were performed (see Table 1), and the number of types of professional breaches accompanying the unnecessary procedures. These variables were first standardized to Z-scores, then aggregated to a summary score and standardized to a T-score (mean = 50, SD = 10). Cluster 1 cases generated the highest mean seriousness score: 54.4 (7.9). When compared to the other clusters using the independent t-test, this mean was significantly (*p* < .05) higher than that for Cluster 3, 45.6 (11.7), and Cluster 5, 47.9 (10.8); it did not differ significantly from Cluster 2, 51.9 (8.2), or Cluster 4, 50.9 (7.9).

Other variables demonstrated discrimination among clusters. Presence of an accomplice was lowest in Cluster 4 (9.1%), highest in Cluster 5 (90.5%), and moderate in Clusters 1, 2, and 3 (44.4%–64.7%), V = .52. Solo practice ownership was low in Clusters 5 (0%) and 4 (18.2%), and moderate in Clusters 1, 2, and 3 (35.3%–55.6%), V = .45. Physicians who engaged in unrelated illegal actions were more prevalent in Clusters 1 (38.1%) and 3 (35.3%), relative to Clusters 2, 4, and 5 (4.8%–11.1%), V = .36. Cluster 2 physicians were least likely to have defrauded Medicare (22.2%), compared to physicians in Clusters 1 and 3–5 (66.7%–90.5%), V = .46.

## Discussion

We identified 79 applicable cases of unnecessary procedures; these represented 100% of cases identified through a systematic literature review of publicly available records that met our inclusion criteria. By coding case characteristics and variables that theoretically provide motive, means or opportunity for the performance of unnecessary procedures, we identified a series of variables that cut across at least 80% of cases. The cases predominantly involved males (96.2%), in non-academic medical practices (92.4%), who were motivated by financial gain (92.4%), practicing in an environment with some type of oversight deficit (80%).

Many of the characteristics of physicians in our sample were similar to national averages. For example, the age of physicians in our sample mirrored the national average with 48% (versus 47% nationally) being older than 49 years [37]. The percentage of physicians who were board certified (70.9) was nearly identical to the national average for physicians who trained outside of the U.S. (70%) and slightly lower than those who trained in the U.S. (77%) [37]. The number of physicians born outside of the U.S. (27.8%) was identical to the national average [38]. Ninety-two percent of cases occurred in non-academic settings; this may roughly reflect national averages: Of those individuals who completed residency training from 2006 through 2015, 16.6% currently hold a full-time faculty appointment at a U.S. MD-granting medical school, [39] but not all physicians complete a residency, and other sources claim that just 7% of U.S. physicians worked in an academic medical center during the time most of our cases occurred [40].

Other variables diverged noticeably from national averages. The number of males (96.2%) was higher than the number working in the field (69%) during the median time cases were reported [41]. The number of physicians working in solo practice was higher than the expected national value (17.7% in our sample versus 13% nationally)—and an even greater number (27%) were sole owners of their practice even when they had practice partners [41]. The percentage of physicians with suspected antisocial personality disorder was much higher than the prevalence in the general U.S. population, which is between 3.9% and 5.8% in men and 0.5% and 1.9% in women [42]; however, it was similar to the prevalence among male prisoners, which studies estimate to be between 35 and 47% [43, 44]. The percentage of physicians who trained outside of the U.S. (40.5%) was also markedly higher than the national average (24.0%) [37, 41]. As a whole, this pattern is consistent with the findings of studies that examined factors associated with being disciplined by a state medical board—being male, not board certified, and graduating from a non-U.S. medical school were all statistically significantly associated with being disciplined [45, 46].

Cases could be divided into typologies primarily by distinguishing those involving physicians with suspected antisocial personalities, by distinguishing specific kinds of problems with oversight (i.e., oversight failures, lack of oversight, and a corrupt moral climate), and a small number of cases involving financial conflicts of interest or poor problem-solving. These typologies provide a key to tailoring recommendations.

Clusters 1 and 5 were the largest and comprised 53% of cases. All but one of these cases involved financial gain as a motive. In cluster 1, 100% involved suspected personality disorders and either a lack or failure of oversight; in cluster 5, 100% of cases included a corrupt moral climate, which invariably involved either others being convicted as part of the scheme or the institution paying fines. These are all straightforward cases of deviant behavior. Given the oversight deficits that accompany such cases, they frequently require whistleblowers, for example, nurses, patients, family members, or other physicians who are visited following complications from the unnecessary procedures. Given that most instances of unnecessary procedures are repeat instances, the most effective way to prevent cases is through early intervention. The strongest predictor of a complaint to a medical board is a past complaint; [24] this pattern starts early, even with complaints against individuals in medical school and residency programs [47, 48]. This suggests that colleagues, nurses, and administrators should be trained to watch for red flags and be empowered to report them in a timely manner, with an assurance of action and freedom from retribution. At this time, few data exist to guide decisions regarding when remediation versus termination or loss of license is appropriate; in the interest of protecting patients, at a minimum, increased observation should accompany remediation efforts following more minor events.

Cluster 4 indicated that it is possible for unnecessary procedures to be performed without any kind of oversight problem. Nevertheless, some form of oversight problem provided opportunity in 80% of cases, making it the most prevalent environmental factor and the second most prevalent MMO variable after financial gain. It played a far more significant role than, say, relationships to industry or treating vulnerable participants. Indeed, some of the most effective interventions aimed at reducing malpractice lawsuits have focused on tracking physician behavior (e.g., attracting unsolicited patient complaints) and providing timely peer feedback (e.g., sharing comparative data on complaints) [49, 50].

Clusters 2 and 3, which were both small, commonly involved financial gain as a motive, but primarily illustrate the potential for relationships to industry or poor skills to contribute to the performance of unnecessary procedures. It is too early to tell whether measures such as the Physician Payments Sunshine Act will serve to reduce unnecessary procedures by empowering patients and colleagues to monitor behavior in light of financial relationships to industry; initial analyses suggest transparency alone is inadequate to manage bias [51]. Clarifying practice guidelines might close the door on some cases because a lack of clarity about what is medically necessary creates space for the performance of unnecessary procedures [17]. By examining cluster 3, we see that gray areas seem to facilitate the performance of unnecessary procedures particularly among those who do not appear to have a personality disorder, which makes sense: ambiguous norms are easier for the average person to violate than norms that clearly protect others from harm. Of all the clusters, this cluster also had the lowest seriousness rating—45.6 versus 54.4 in cluster 1, which included only physicians with suspected personality disorders. Ambiguous norms played no role in any of the cases involving poor problem-solving or a lack of skills, which reinforces the notion that ambiguous norms create space for normal physicians to act upon financial gain, rather than simply make incompetent decisions. At the same time, interventions involving physician audit and feedback have successfully reduced unnecessary cardiac tests; such an approach might also succeed in reducing rates of unnecessary procedures with regard to physicians who are trying to practice medicine with integrity [52]. Ideally, such audits would be conducted by peer review committees or institutions; as Buck notes, when physicians and healthcare institutions fail to self-regulate, the result is regulation and oversight by federal prosecutors and other non-physician oversight bodies [53]. Hospital boards may have a significant role to play in treating unnecessary procedures as a matter of patient safety and incorporating prevention into their missions [54].

Overall, the cases point to limitations of our current abilities to detect promptly and decisively respond to the occurrence of unnecessary procedures. Ninety-two percent of cases persisted for more than 2 years; 53% persisted for more than 5 years. Numbers of patients were high, and more than half of cases (53.2%) involved billing more than $1 million for the unnecessary procedures. Nearly half of all physicians in our sample (48.1%) performed unnecessary procedures in more than one environment, which might have increased the potential for oversight and intervention. We found evidence that in at least 35% of cases there was a missed opportunity to blow the whistle, and that in 25% of cases a whistleblower was ignored while the behavior persisted. These are likely underestimates of the actual numbers, because it is impossible to determine how frequently colleagues were highly suspicious of the invasive procedures but chose not to intervene. Given the number of patients harmed and the amount of money billed, it is noteworthy that only 55.7% of physicians lost their license and 58.2% discontinued practicing medicine long-term. Medical boards should be empowered and expected to revoke licenses when a physician poses a significant threat to patient safety.

Medicare was billed for unnecessary procedures in 73.4% of cases. While it is not surprising that the federal government frequently pursues fraud charges when billed for unnecessary procedures, this percentage is high considering that Medicare covers only 16.9% of the U.S. population [55]. It is worth investigating further whether unnecessary procedures occur at a similar rate across third party payer plans, but is simply under-investigated or underreported when it does not involve Medicare. If so, this would suggest that private insurers could play a bigger role in identifying and reporting unnecessary procedures; however, this might require access to overall performance data on physicians, as any one private insurer may have difficulty identifying patterns in a physician’s overall procedures.

### Limitations

We identified 79 applicable cases of unnecessary invasive procedures; these represented 100% of cases identified through systematic literature review. Given the severe limitations of available information, however, we did not have access to detailed case information for the many additional cases that involve malpractice charges and are settled out of court, nor for many of the cases reported to the National Practitioners Database, which de-identifies data and provides information on few variables relevant to determining MMO [32]. Moreover, our approach is likely to underestimate the prevalence of variables in some cases because it was dependent upon the amount of information included in public records and published articles.

## Conclusions

This study contributes to efforts to ensure the safety of patients and to protect the fiduciary relationship of physicians to patients. Unnecessary procedures harm the healthcare system by wasting funding; it also directly harms patients physically, causes unnecessary worry, and costs patients time and money. This study provides data that can inform efforts to prevent the occurrence and recurrence of unnecessary procedures.

There are few demographic red flags for the performance of unnecessary procedures. The following variables characterized roughly half (40–60%) of cases: having a suspected personality disorder, training outside of the U.S., being at least 50 years old, practicing as employees, and working in large practices. This means that, conversely, about half had no suspected personality disorder, trained in the U.S., were under 50 years old, and were self-employed or worked in smaller practices. As noted above, some of these frequencies are greater than expected and accordingly could be viewed as risk factors. However, the performance of unnecessary procedures appears best understood in terms of MMO variables rather than the demographic variables: It is a behavior that is usually motivated by financial gain within a fee-for-service model and occurs in settings that have oversight problems (including a lack of oversight, oversight failures, or a corrupt moral climate). In some cases, it is due to poor professional decision-making or skills, usually also accompanied by some kind of oversight problem. Accordingly, preventive efforts should focus on clarifying the standard of care where guidelines are unclear and widely disseminating guidelines: Gray areas provide fertile ground for unnecessary procedures. Preventing the recurrence of unnecessary procedures requires early detection by peers and institutions, and decisive action by medical boards or federal prosecutors in order to protect the safety of patients.

## Abbreviations

FCOI: Financial conflict of interest
MMO: Motive, means, and opportunity

## Footnotes

1 The following terms were used in the search strategy: ((Physician OR Doc OR Doctor OR Dr. OR Surgeon OR Psychiatrist OR Pediatrician OR!Surgeon OR Oncologist) W/20 (Charg! OR Accus! OR Convict! OR Revok! OR Suspen! OR Disciplin! OR Fine! OR Sanction! OR Probation OR Censure! OR Arrest! OR Guilty OR Settle! OR Agreement OR Sue! OR!Suit!)) AND ((Unneeded OR Unnecessary OR Unwarranted OR Fraud!) w/2 (Surger! OR Operat! OR Test! OR Lab! OR Treatment OR Inject! OR Procedure OR Imag! OR Diagnos! OR Stent! OR Chemo! OR!Fusion!))
